# Influence of Asymmetrical Eruption and Impaction Angulation of the Wisdom Teeth on the Craniofacial Morphology

**DOI:** 10.7759/cureus.101112

**Published:** 2026-01-08

**Authors:** Akinori Moroi, Karen Gomi, Reiji Kojima, Riku Kohara, Yusuke Kurosawa, Kunio Yoshizawa, Koichiro Ueki

**Affiliations:** 1 Department of Oral and Maxillofacial Surgery, Division of Medicine, Interdisciplinary Graduate School, University of Yamanashi, Chuo-shi, JPN; 2 Department of Epidemiology and Environmental Medicine, School of Medicine, University of Yamanashi, Chuo-shi, JPN

**Keywords:** asymmetry, cephalometric analysis, craniofacial morphometry, three-dimensional computed tomography, wisdom teeth

## Abstract

Objectives: Congenital absence of the wisdom teeth is correlated with the jaw morphology; however, no study has investigated the correlation between the direction of eruption or impaction of the wisdom teeth and jaw morphology. This study aimed to investigate whether differences in the direction of eruption or impaction between the bilateral wisdom teeth affect the jaw morphology.

Methods: The study included patients with bilateral wisdom teeth who underwent computed tomography imaging prior to wisdom-tooth extraction. The direction of eruption or impaction of the wisdom teeth was evaluated, and patients were divided into the same-angle and different-angle groups based on the directions of eruption or impaction of the bilateral mandibular wisdom teeth. Lateral and frontal cephalometric images were reconstructed using computed tomography data, and three-dimensional measurements of the mandibular ramus and body were compared between the two groups.

Results: The same- and different-angle groups included 70 and 81 patients, respectively. Lateral cephalometric analysis revealed significant differences in angle ANB (p=0.001), the incisor-mandibular plane angle (p=0.032), the difference in the Menton-Anterior notch distance (p=0.036), and the maxillo-mandibular midline angle (p=0.006) between the groups. However, three-dimensional measurements revealed no significant differences between the groups.

Conclusions: This study showed that patients with dissimilar directions of eruption or impaction of the bilateral mandibular wisdom teeth tend to have mandibular asymmetry. The findings in this study may facilitate the diagnosis of jaw deformities based on an analysis of the direction of eruption or impaction of the wisdom teeth.

## Introduction

Jaw deformities are characterized by an abnormal maxillary or mandibular morphology, resulting in malocclusion and dysfunction. Several studies have reported correlations between jaw deformity and temporomandibular joint disorders and the perioral muscles [1-3]. Therefore, early diagnosis and intervention are necessary to improve maxillofacial health. Conventionally, jaw deformities are diagnosed based on cephalometric findings [4]. However, a more accurate diagnosis based on three-dimensional (3D) computed tomography (CT) has recently been reported [5]. Nevertheless, screening tests for jaw deformities are lacking, and opportunities for diagnosis or treatment of jaw deformities are missed in patients with few subjective symptoms.

Few studies have reported a correlation between the maxillary and mandibular morphology and the congenital absence of wisdom teeth [6,7]. Particularly, in patients with congenitally missing wisdom teeth, the maxilla and mandible tend to be smaller [8]. Furthermore, the absence of wisdom teeth is strongly correlated with craniofacial development, and wisdom teeth play an essential role in the evolutionary miniaturization of the craniofacial region [9]. The wisdom teeth can erupt or be impacted in various ways, as classified by Pell and Gregory. Although the exact reason for wisdom tooth impaction is unknown, several theories have been proposed, including jaw-size reduction, eruption path/direction, environmental/genetic factors, and modern lifestyle/diet [10,11]. However, studies evaluating the correlation of asymmetrical eruption or impaction of the wisdom teeth with maxillofacial morphology are lacking.

Clarifying the relationship between impacted wisdom teeth and maxillofacial morphology may aid in diagnosing jaw deformity. Therefore, this study aimed to investigate whether differences in the direction of eruption or impaction between the bilateral wisdom teeth affect craniofacial morphology. The null hypothesis was that asymmetrical eruption or impaction of wisdom teeth is not related to jaw morphology.

## Materials and methods

The protocol of this retrospective study was approved by the Ethics Committee of University of Yamanashi Hospital (approval number: 2910). The researchers followed the guidelines set out in the Declaration of Helsinki throughout this study, and written informed consent for inclusion in the study was obtained from all participants.

Patients

Patients who visited the Department of Oral and Maxillofacial Surgery at University of Yamanashi Hospital between April 1, 2021, and July 31, 2024, for wisdom tooth extraction were included. The inclusion criteria were (1) CT performed for wisdom-tooth extractions and (2) presence of the bilateral mandibular wisdom teeth. The exclusion criteria were (1) congenital disorders related to the maxillofacial region, (2) congenitally missing teeth, (3) history of orthodontic treatment, (4) age <21 years, and (5) diagnosis of jaw deformity.

CT data

Prior to wisdom-tooth extraction, CT was performed using Aquilion One (Toshiba Medical Systems Corp., Tochigi, Japan) using the following parameters: slice thickness, 0.5 mm; voltage, 80-120 kV; current, 280 mA; speed, 0.35 s/rotation; pitch factor, 0.641; algorithm, FC21.

The original data were converted to DICOM format and reconstructed using Synapse Vincent (FUJIFILM Corporation, Tokyo, Japan) to create a 3D CT model with a setting that visualized areas with ≥500 Hounsfield units. The 3D model was established such that the Frankfurt and horizontal planes were parallel.

Wisdom-tooth angle

**Figure 1:**
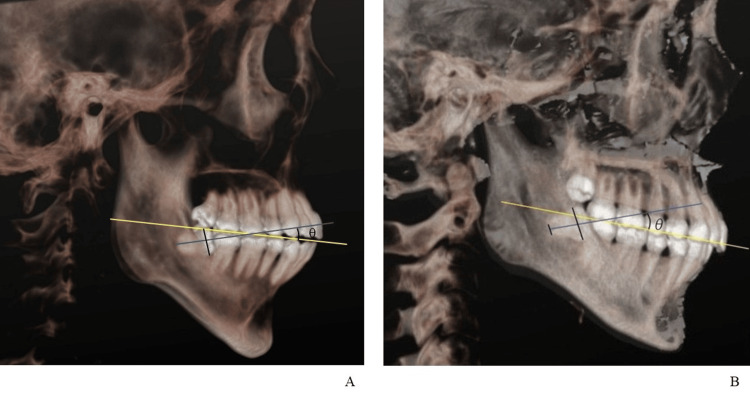
Wisdom-tooth angle measurement A: Single-rooted teeth, B: Multi-rooted teeth Yellow line: The line connecting the incisal edge of the mandibular central incisor and the distobuccal cusp of the second molar is defined as the occlusal plane. Blue line: The direction of the wisdom teeth is defined as the line connecting the midpoint of the maximum prominence of the crown and the midpoint of the root apex of the wisdom tooth. Wisdom-tooth angle (θ): The angle between the yellow and blue lines.

On the two-dimensional (2D) screen orthogonal to the straight line connecting the upper margins of the left and right ear foramina, the midpoint of the line connecting the maximum prominence of the wisdom-tooth crown and the midpoint of the root apex was defined as the direction of the wisdom tooth. In single-rooted wisdom teeth, a straight line connecting the farthest end of the root and the midpoint of the maximum prominence of the wisdom-tooth crown was defined as the direction of the wisdom tooth, and in multi-rooted wisdom teeth, a straight line connecting the midpoint of the roots and the midpoint of the maximum prominence of the wisdom-tooth crown was defined as the direction of the wisdom tooth. Further, the angle between a line joining the distal cusp of the mandibular right second molar and the incisal edge of the mandibular right central incisor and the direction of the wisdom tooth was defined as the wisdom-tooth angle (Figure 1). Thereafter, the image was rotated by 180° in the horizontal plane, and measurements for the opposite side were made in the same manner. Two authors (M.A. and G.K.) performed all measurements thrice, and the mean value was considered the wisdom-tooth angle.

Grouping

Patients with a difference in inclination of the left and right wisdom teeth of <22.5° and ≥22.5° were classified into the same-angle and different-angle groups, respectively. The cutoff for wisdom tooth inclination of 22.5° was set based on the definitions of the horizontal, vertical, and mesial inclination positions according to Winter's classification. The angle between the wisdom-tooth axis in the horizontal and vertical positions was set to 90°. The angle between the tooth axes in the horizontal or vertical and mesioangular positions was set to 45°. Based on this setting, a difference of 22.5° resulted in a different category according to Winter's classification and was set as the standard value [12].

Three-dimensional CT measurement

The lengths of the mandibular ramus and body were measured in 3D. Measurements were made on the 3D screen orthogonal to the straight line connecting the upper margin of the left and right ear foramina.

**Figure 2:**
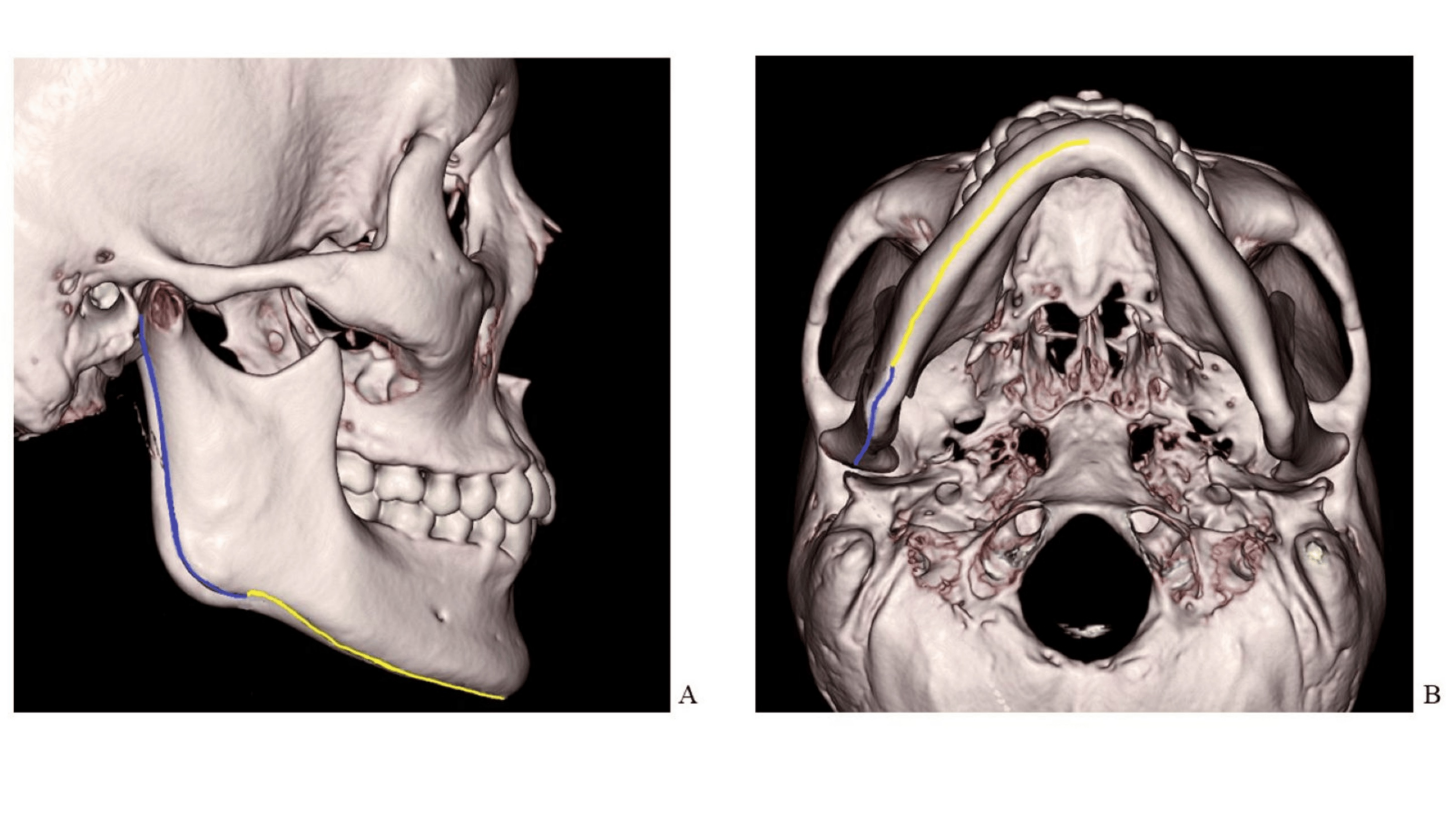
Three-dimensional measurement A: Lateral image; B: Image rotated 90° downward from the lateral image. Blue line: Length of the mandibular ramus. Yellow line: Length of the mandibular body.

The length of the mandibular ramus was measured along the posterior border from the most posterior point of the condylar head to the lowest point on the mandibular angle. The length of the mandibular body was measured along the inferior border from the lowest point on the mandibular angle to the point of intersection of a line connecting the mental spine and mandibular body (Figure 2). After obtaining the measurements on the right side, the image was rotated by 180° in the horizontal plane, and measurements for the left side were obtained in the same manner.

Cephalometric analysis

The original imaging data were converted to DICOM format and reconstructed using Simplant version 18 (Dentsply Sirona, Charlotte, NC, USA) to create lateral and frontal cephalometric radiographs. Cephalometric analysis was performed using Cephalometric A to Z (Yasunaga Computer Systems Co., Inc.).

**Figure 3:**
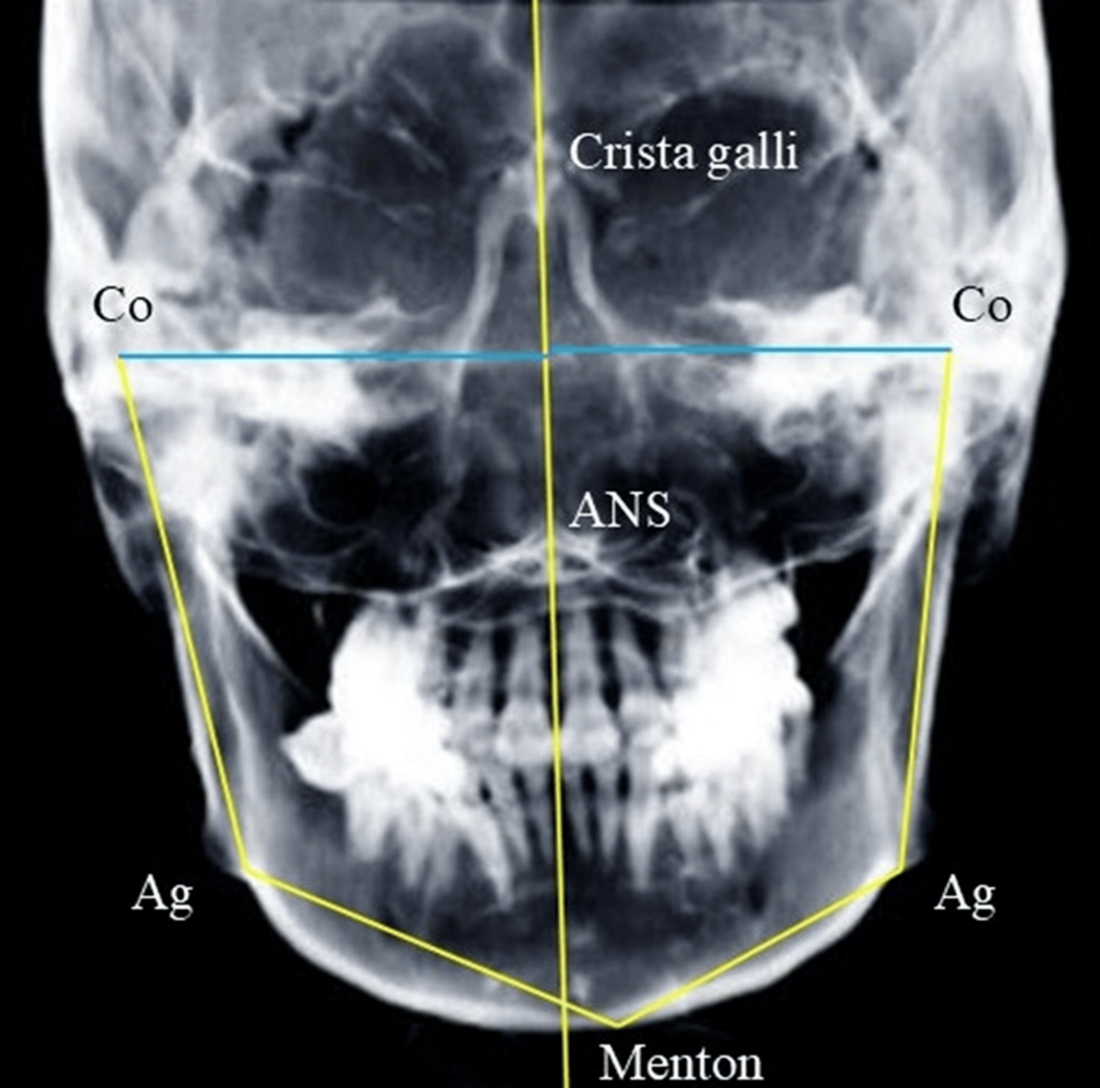
Measurements on the frontal cephalogram Co: condylion, Ag: anterior notch, ANS: anterior nasal spine Co distance: Distance between Co and the line connecting crista galli and ANS

**Figure 4:**
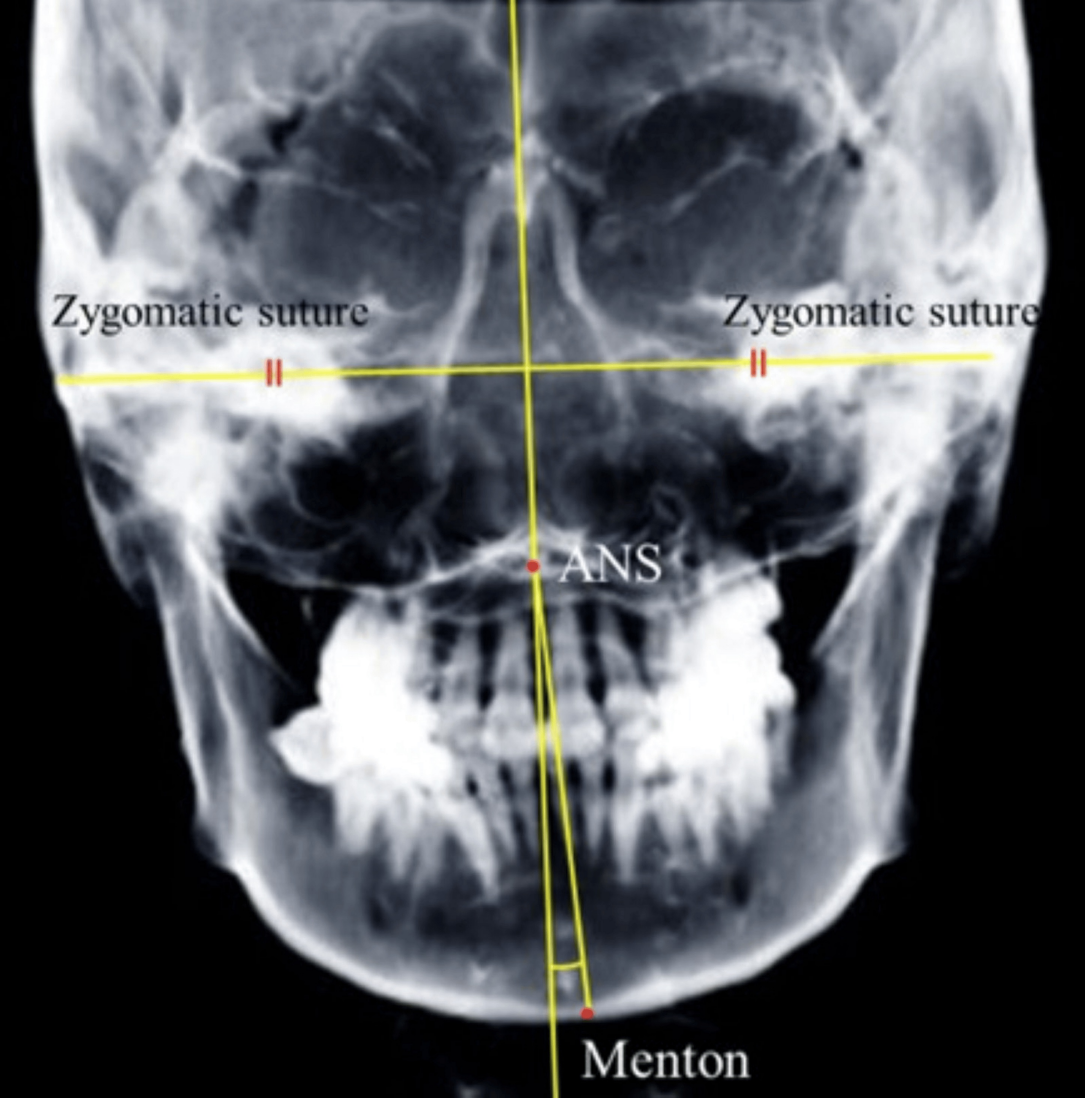
Definition of the Mx-Md midline angle The angle between a line perpendicular to the line connecting the bilateral zygomatic sutures and passing through the ANS and a line connecting the ANS and Menton is defined as the Mx-Md midline angle. Mx-Md: maxillomandibular; ANS: anterior nasal spine

The following variables were measured on lateral cephalograms: angles SNA, SNB, and ANB; Frankfurt’s mandibular plane angle (FMA), Frankfurt’s mandibular incisor angle (FMIA), incisor-mandibular plane angle (IMPA), gonial angle, and occlusal-plane angle. To evaluate symmetry, the following variables were measured on frontal cephalograms, and the difference between the values on the left and right sides was calculated: distance between the condylion (Co) and the line connecting crista galli and anterior nasal spine (ANS), distance between the anterior notch (Ag) and the line connecting crista galli and ANS, Ag angle, and the angle between the line connecting condylion and Ag and the line connecting Menton (Me) and Ag (Figure 3). The maxillo-mandibular (Mx-Md) midline angle was measured on frontal cephalograms, based on previous studies [13,14]. The angle between the line perpendicular to the line connecting the bilateral zygomatic sutures and passing through the ANS and the line connecting the ANS and Me was defined as the Mx-Md midline angle (Figure 4). The side on which the Me was deviated was defined as the deviated side, and the opposite side as the non-deviated side. Two authors (M.A. and G.K.) performed all measurements twice, respectively, and the mean value was used for analysis.

A previous study used a 2.5° Mx-Md line angle as the standard to distinguish between symmetry and asymmetry [15]. This criterion was applied to assess the proportion of patients with asymmetry in both groups.

Statistical analysis

Data were statistically analyzed using IBM Corp. Released 2019. IBM SPSS Statistics for Windows, Version 25. Armonk, NY: IBM Corp. Differences in the wisdom-tooth angles on the right and left sides and age were compared between the two groups using the Mann-Whitney U test. Cephalometric variables and 3D measurements were compared between the different- and same-angle groups using the Mann-Whitney U test. Furthermore, a χ-square test was used to compare the proportion of patients with an Mx-Md midline angle >2.5° and sex between the same- and different-angle groups. Statistical significance was set at p<0.05.

## Results

Patients

**Figure 5:**
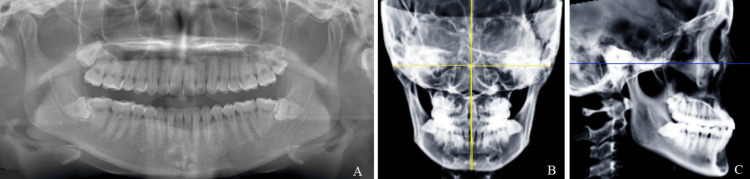
Representative images of the same-angle group A: Panoramic radiograph. B: Mx-Md midline angle is measured in the frontal cephalogram. C: Lateral cephalogram. Blue line: Frankfort plane

**Figure 6:**
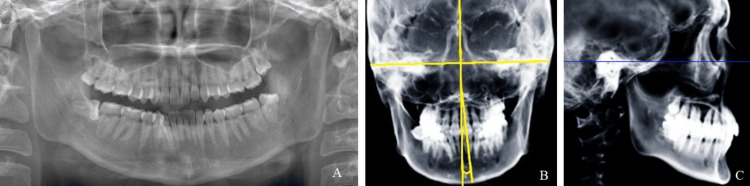
Representative images of the different-angle group A: Panoramic radiograph. B: Mx-Md midline angle is measured in the frontal cephalogram. C: Lateral cephalogram. Blue line: Frankfort plane

A total of 151 patients were included in this study. Of these, 81 patients (mean age, 31.3±9.1 years; M:F, 26:55) were included in the different-angle group, and 70 patients (mean age, 31.4±13.1 years; M:F, 29:41) were included in the same-angle group (Figures 5, 6). No statistically significant difference in the sex ratio was identified between the two groups (Table 1). Therefore, we believe that age and sex differences between the two groups had little effect on the evaluation of the mandibular morphology.

Wisdom-tooth angle

The difference in the inclinations of the left and right wisdom teeth in the same- and different-angle groups was 5.5° and 42.4°, respectively, and it was significantly different between the groups (p<0.001) (Table 1). In the same-angle group, the directions of eruption or impaction of the bilateral mandibular wisdom teeth were almost the same, whereas in the different-angle group, the median difference in the directions of eruption and impaction of the bilateral mandibular wisdom teeth was approximately 40°.

**Table 1:** Distribution of sex, age, and difference in the wisdom-tooth eruption or impaction angles between the right and left sides in the same- and different-angle groups

	Same-angle group	Different-angle group	p-value
Male	29(41.4％)	26(32.1%)	0.141
Female	41(58.6％)	55(67.9%)	
Age	31.4±13.1	31.3±9.1	0.225
Difference between wisdom-tooth angles between the right and left side (degree)	5.5	42.4	<0.001

Cephalometric analysis

Angle ANB (p=0.001), the incisor-mandibular plane angle (IMPA) (p=0.032), the difference in Ag distance (p=0.004), the difference in the Me-Ag distance (p=0.036), and the Mx-Md midline angle (p=0.006) were significantly different between the groups (Table 2). Cephalometric analysis suggested that the difference in the direction of eruption or impaction of the mandibular third molars affected the maxillo-mandibular morphology in the anterior region and the symmetry of the craniofacial region.

**Table 2:** Cephalometric analysis variables were compared between the different- and same-angle groups using the Mann–Whitney U test Cephalometric variables were compared between the different- and same-angle groups using the Mann–Whitney U test. FMA: Frankfurt’s mandibular plane angle; FMIA: Frankfurt’s mandibular incisor angle; IMPA: incisor–mandibular plane angle; Mx-Md: maxillo-mandibular; Co: condylion; Ag: anterior notch; Me: Menton.

	Medial value		95% confidence interval for the difference between the two groups	p-value
	Same angle group	Difference angle group		
SNA (degree)	81.1	81.8	-2.9 - 0.4	0.146
SNB (degree)	78	77.7	-0.9 - 2.2	0.427
ANB (degree)	2.6	3.9	-2.1 - -0.4	0.001
FMA (degree)	27.3	28.1	-3.3 - 1.2	0.525
IMPA (degree)	96.9	93.5	0.1 - 4.4	0.032
FMIA (degree)	56.8	57.2	-3.7 - 1.6	0.494
Gonial angle (degree)	124.8	125.3	-2.8 - 1.8	0.932
Occlusal plane (degree)	8.3	8.7	-2.8 - 0.4	0.092
Difference Co distance (mm)	1.21	1.24	-1.3 - 0.4	0.551
Difference Ag distance (mm)	0.4	1.26	-2.2 - -0.3	0.004
Difference Ag angle (degree)	0.13	0.92	0.1 - 2.7	0.065
Difference Co - Ag (mm)	0.28	0.6	-1.4 - 0.2	0.372
Difference Me - Ag (mm)	1.12	1.62	-2.1 - -0.5	0.036
Mx-Md midline angle (degree)	1.7	2.15	-1.7 - -0.3	0.006

Specifically, the proportion of patients with an Mx-Md midline angle greater than 2.5° was 27.1% (19/70) and 44.4% (36/81) in the same- and different-angle groups, respectively, and the difference was statistically significant (p = 0.028). However, even in the group with a difference in the wisdom-tooth angle on the left and right sides, more than half did not have a clinically significant asymmetry.

Three-dimensional CT measurement

In the same- and different-angle groups, the lengths of the mandibular ramus and body were not significantly different between the deviated and non-deviated sides (Table 3). These findings indicate that the difference in the direction of eruption or impaction of the bilateral mandibular third molars does not affect the difference in the mandibular length on the left and right sides.

**Table 3:** Comparison of three-dimensional computed tomography measurements between the deviated and non-deviated sides in both groups using the Mann-Whitney U test

	Same - angle group		p-value	Different - angle group		p-value
	Deviation side	Non-deviation side		Deviation side	Non-deviation side	
Ramus length (mm)	44.4	46.8	0.126	46.5	47.1	0.723
Mandibular body length (mm)	86.8	87.8	0.440	88.7	88.2	0.963

## Discussion

The wisdom teeth begin to form at approximately 3-4 years of age. Calcification of the wisdom-tooth crowns begins at approximately 7-10 years, and they erupt at approximately 17-21 years of age. Mandibular growth peaks at approximately eight years and ends at approximately 18 years [16,17]. Thus, the periods of mandibular growth and wisdom teeth development overlap, and the presence of wisdom teeth may influence the mandibular morphology [9]. This study showed that patients with different eruptions or impaction directions of the bilateral mandibular wisdom teeth tend to have asymmetrical jaws. These results suggest a close relationship between wisdom-tooth maturation and jaw morphology, which is consistent with the findings in previous studies on the effect of congenital absence of wisdom teeth on the jaw morphology. The different-angle group had a significantly higher proportion of patients with an Mx-Md midline angle ≥2.5°, a criterion used in previous studies for classifying patients with asymmetry. However, in this group, only 45% of patients demonstrated an Mx-Md midline angle ≥2.5° [15,18]. These findings suggest that craniofacial asymmetry is likely to occur when the directions of eruption or impaction of the bilateral mandibular wisdom teeth differ. However, differences in the angles of the left and right wisdom teeth do not necessarily indicate asymmetry in maxillofacial morphology, and they may be one of the findings in patients with jaw asymmetry.

Panoramic radiography allows visualization of the entire upper and lower jaws, and because of the low radiation exposure, it is used as a screening test during the first dental visit to diagnose caries, alveolar-bone volume, and maxillary or mandibular lesions [19]. Differences in the direction of eruption or impaction of the bilateral wisdom teeth can be diagnosed using panoramic radiography. Therefore, the results of this study indicate that panoramic imaging is useful not only for screening for general dental disease but also for clinical detection of jaw asymmetry based on asymmetric eruption or impaction of the wisdom teeth.

Various factors influence the direction of eruption or impaction of the wisdom teeth. The retromolar space is the most important factor affecting the direction of eruption or impaction of the wisdom teeth, and an increase in the retromolar space by 1 mm reduces the probability of impacted wisdom teeth by 30% [20]. However, several studies have reported no correlation between the retromolar space and the eruption or impaction of the wisdom teeth [21,22]. Although the correlation between the eruption of the wisdom teeth and mandibular morphology has been assessed using various modalities and evaluation methods, the findings remain inconclusive. Cephalometric analysis using 2D images has been used for some time and is a useful method for measuring facial features because it involves several measurement items. However, slight errors resulting in reduced reproducibility may occur when obtaining lateral or frontal photographs. Therefore, in this study, lateral and frontal cephalometric images were reconstructed from CT data to prevent photographic errors and used for measurements. In addition, the total mandibular length was measured in 3D to accurately measure local areas. Two-dimensional images are more effective for providing an overview, whereas 3D images are more suitable for precise measurements of specific parts [23]. Therefore, in this study, jaw deformities were identified on 2D images, whereas no deformities were observed on 3D images. In this study, 3D measurements were exclusively performed on the mandible; however, it is posited that the absence of observed differences in the mandibular morphology is attributable to the occurrence of asymmetry throughout the maxillofacial region. Morphological abnormalities of the jaw can be accurately diagnosed through the application of both 2D and 3D examinations. Therefore, jaw-morphology evaluation should include a comprehensive assessment using both 2D and 3D images obtained using multiple modalities.

In this study, angles ANB and IMPA differed according to the direction of wisdom tooth eruption or impaction. Recent reports have indicated a trend for mandibular bone reduction associated with the absence of wisdom teeth [8]. Similarly, the directions of eruption and impaction of the wisdom teeth may influence mandibular morphology. In this study, angles SNA and SNB and the gonial angle were not significantly different between the groups. Therefore, the morphological abnormalities of the maxillofacial region indicated by angle ANB and IMPA measurements were believed to be localized phenomena. Asymmetrical eruption or impaction of the wisdom teeth likely exerts a limited effect on this craniofacial region.

This study had some limitations. First, because this was a retrospective study, selection bias could not be ruled out. Therefore, we strictly controlled the period of visits of the patients and the inclusion and exclusion criteria to prevent bias. Second, various factors, such as ethnicity or dietary habits, have been suggested as the cause of impacted wisdom teeth; however, this study did not examine these factors. Therefore, these potential factors may have influenced the occurrence of impacted wisdom teeth. Third, the criterion for categorizing the same-angle and difference-angle groups was set at 22.5° to ensure adequate statistical power (post-hoc power measurements showed a power of 0.757 for the Mx-Md midline angle) and to elucidate the asymmetry in the direction of eruption or impaction of the wisdom teeth. It is important to note that study findings may vary based on the classification criteria, necessitating careful consideration in this regard. Fourth, the mechanism through which the wisdom teeth were correlated with the maxillofacial morphology was unclear. Therefore, whether the relationship between the wisdom teeth and jaw morphology was direct or indirect remains uncertain. Future studies should explore the relationship between the wisdom teeth and maxillo-mandibular morphology using different approaches.

## Conclusions

This study demonstrated that differences in the direction of eruption or impaction of the bilateral mandibular wisdom teeth may impact jaw symmetry. Therefore, assessment of the bilateral mandibular wisdom teeth using panoramic radiographs may serve as a screening method for detecting facial asymmetry. Furthermore, our findings indicate a correlation between the direction of eruption of the third molars and craniofacial morphology. Based on this finding, we hypothesize that the timing of craniofacial alterations may align with the developmental stage of third-molar formation. We hope that future longitudinal studies will clarify this hypothesized relationship between the eruption pattern of third molars and craniofacial morphology.
